# Changes in myocardial blood flow in a canine model of left sided breast cancer radiotherapy

**DOI:** 10.1371/journal.pone.0291854

**Published:** 2023-09-28

**Authors:** Oi-Wai Chau, Omar El-Sherif, Matthew Mouawad, Jane M. Sykes, John Butler, Heather Biernaski, Robert deKemp, Jennifer Renaud, Gerald Wisenberg, Frank S. Prato, Stewart Gaede

**Affiliations:** 1 Department of Medical Biophysics, Western University, London, Ontario, Canada; 2 Department of Physics and Radiation Oncology, London Regional Cancer Program, London, Ontario, Canada; 3 Mayo Clinic, Rochester, Minnesota, United States of America; 4 Thames Valley Veterinary Services, London, Ontario, Canada; 5 Lawson Health Research Institute, London, Ontario, Canada; 6 National Cardiac PET Centre, University of Ottawa Heart Institute, Ottawa, Ontario, Canada; 7 Division of Cardiology, London Health Sciences Centre, London, Ontario, Canada; Emory University, UNITED STATES

## Abstract

**Background:**

Left-sided breast cancer patients receiving adjuvant radiotherapy are at risk for coronary artery disease, and/or radiation mediated effects on the microvasculature. Previously our laboratory demonstrated in canines with hybrid ^18^FDG/PET a progressive global inflammatory response during the initial one year following treatment. In this study, the objective is to evaluate corresponding changes in perfusion, in the same cohort, where resting myocardial blood flow (MBF) was quantitatively measured.

**Method:**

In five canines, Ammonia PET (^13^NH_3_) derived MBF was measured at baseline, 1-week, 1, 3, 6 and 12-months after cardiac external beam irradiation. MBF measurements were correlated with concurrent ^18^FDG uptake. Simultaneously MBF was measured using the dual bolus MRI method.

**Results:**

MBF was significantly increased at all time points, in comparison to baseline, except at 3-months. This was seen globally throughout the entire myocardium independent of the coronary artery territories. MBF showed a modest significant correlation with ^18^FDG activity for the entire myocardium (r = 0.51, p = 0.005) including the LAD (r = 0.49, p = 0.008) and LCX (r = 0.47, p = 0.013) coronary artery territories.

**Conclusion:**

In this canine model of radiotherapy for left-sided breast cancer, resting MBF increases as early as 1-week and persists for up to one year except at 3-months. This pattern is similar to that of ^18^FDG uptake. A possible interpretation is that the increase in resting MBF is a response to myocardial inflammation.

## Introduction

It is projected that in 2021, breast cancer will account for 25% of the total yearly female cancer incidence in Canada [1]. Advances in adjuvant radiation therapy of the breast improves both local control and overall survival [2, 3]. However, patients with left-sided breast cancer are at a greater risk for the later development of radiation-mediated effects on the heart, including effects on the major coronary arteries, as well as vasculature due to the proximity of the heart to the radiation beam [4]. A worldwide systematic review done by Drost et al. on whole breast radiotherapy studies after 2014, reported a 3.6 Gray (Gy) total mean whole heart dose based on 84 left-sided breast cancer studies without breathing control and a lower mean heart dose of 1.7 Gy from 65 regimens with breathing control [5]. The left anterior descending artery located in the anterior region of the heart, which is closer to the left breast, receives a substantially higher mean dose of 12.4 Gy [5]. Darby et al. has reported a linear relationship between major coronary events such as myocardial infarction and death from ischemic heart disease and radiation dose without a threshold (7.4% per Gy mean heart dose) with the incidence increasing with the latency time out to fifteen plus years post RT [6]. Several studies have reported myocardial perfusion deficits following radiotherapy (RT) for left-sided breast at 6 or more months, detected using mainly single photon emission tomography (SPECT). However without measurements performed prior to 6 months, any early postulated effects of radiation on myocardial blood flow and/or metabolism may be related to cardiac conditions that existed prior to RT for left breast cancer [7–11]. Rasmussen et al. reported no differences in MBF between irradiated and non-irradiated myocardium using ^13^N ammonia (^13^NH_3_) PET imaging of breast cancer patients at an average of 7 years post-irradiation [12]. Up to this point, no serial longitudinal MBF assessments have been done which include early post treatment timepoints (i.e., before 6 months post RT) except for a single canine study by Yan et al, [13] which studied animals at 3 time points (months 3, 6, and 12 after radiation. However the clinical relevance of the findings are uncertain, as in that study, the single fraction dose used was 20 Gy which far exceeds the single dose equivalent of a typical clinical RT protocol for left breast cancer. Hence, as the early effects of radiation remain unknown, given the importance of this issue clinically, we wished to assess and monitor the cardiac response to irradiation longitudinally, to look for effects that may manifest from one week to one year post RT for left breast cancer. In a canine model of left-sided breast cancer RT, we measured the changes in myocardial blood flow post cardiac irradiation using both ^13^NH_3_ PET and dynamic contrast enhanced MRI (DCE-MRI) with semi-quantitative measurements done concurrently using hybrid PET/MRI. These semi-quantitative measurements of blood flow were compared and each correlated to the progression of cell-mediated inflammation previously determined using fluorodeoxyglucose (^18^FDG). Measurements performed at 1-week, 1-month, 3-months, 6-months and 12-months in five canines were compared to baseline values.

### Aim

Through the measurement of myocardial blood flow, we will assess the effect of radiation on perfusion in canines from one week to one year in a clinically relevant model similar to patients exposed to left-sided breast cancer RT. We will correlate any changes in perfusion with our previously reported changes in ^18^FDG uptake reflective of inflammation.

## Method

In five adult female, bred-for-research hounds (21–26 kg), cardiac perfusion and inflammation imaging was performed on a hybrid PET/MRI system (Biograph mMR; Siemens AG). The study was approved by the Animal Care Committee of Western University (Protocol 2014–005). All animals at the start of the study were at 1 year old and were anesthetized during imaging and irradiation using propofol (4–6 mg/kg) and maintained with 2% isoflurane. Cardiac perfusion imaging was performed at baseline, 1-week, 1-month, 3-months, 6-months and 12-months following focused cardiac external beam irradiation. The same animal cohort that was used in this perfusion study was used in our previously published ^18^FDG study [14] in which all animals underwent suppression of glycolysis by cardiomyocytes, which included diet, fasting, injection of heparin and infusion of intralipid. Note that we have previously shown that in the canine the most effective and reproducible method to suppress glycolysis by cardiomyocytes is the use of both heparin and intralipid [15]. The injection of heparin and the infusion of intralipid were only applied after the MBF data acquisition was completed. Rate pressure product (RPP) of each animal at each imaging timepoint was determined from the heart rate and systolic blood pressure measured using the physiologic ECG and respiratory unit and the wireless pulse sensor connected to the scanner (Siemens AG).

### Radiation delivery

A fast-helical CT and a contrast enhanced CT (discovery VCT, GE Healthcare) were performed on all animals for radiation treatment planning. Manual contouring of the heart, left ventricle (LV), left circumflex (LCX) and the left anterior descending coronary artery (LAD) perfusion regions were performed on the contrast enhanced CT and overlaid on the fast-helical CT for radiation treatment planning. All animals were irradiated with a TrueBeam linear accelerator (Varian Medical Systems) with the biological equivalent LAD dose for a left-sided breast patient in a single fraction [14, 16]. An α/β ratio of 2.5 Gy was used to convert the multi-fractionated scheme (~30 Gy in 25 fractions prescribed to a focal point at the LAD) to a single ~9 Gy in one fraction [14]. The treatment plans consisted of two, 180°, 6MV photon arcs and deliberately focused on to the myocardial region supplied by the LAD while intentionally avoiding the basal anterolateral portion of the LV and the LCX artery itself in order to compare cardiac function in irradiated vs. minimally-irradiated segments. Dose was calculated using the adaptive convolve dose algorithm implemented on our Pinnacle [3] treatment planning system (Phillips Radiation Oncology Systems). The mean doses delivered to the whole heart (1.7 Gy), LV (2.7 Gy) and the coronary arteries (LAD (5.5 Gy) and LCX (1.1 Gy)) are the typical values observed in left-sided breast cancer radiotherapy patients [5]. The average dose delivered to the myocardium regions supplied by LAD was 2.4 Gy, by LCX was 2.4 Gy and within the overlap regions was 3.1 Gy.

### Imaging

**Fig 1:**
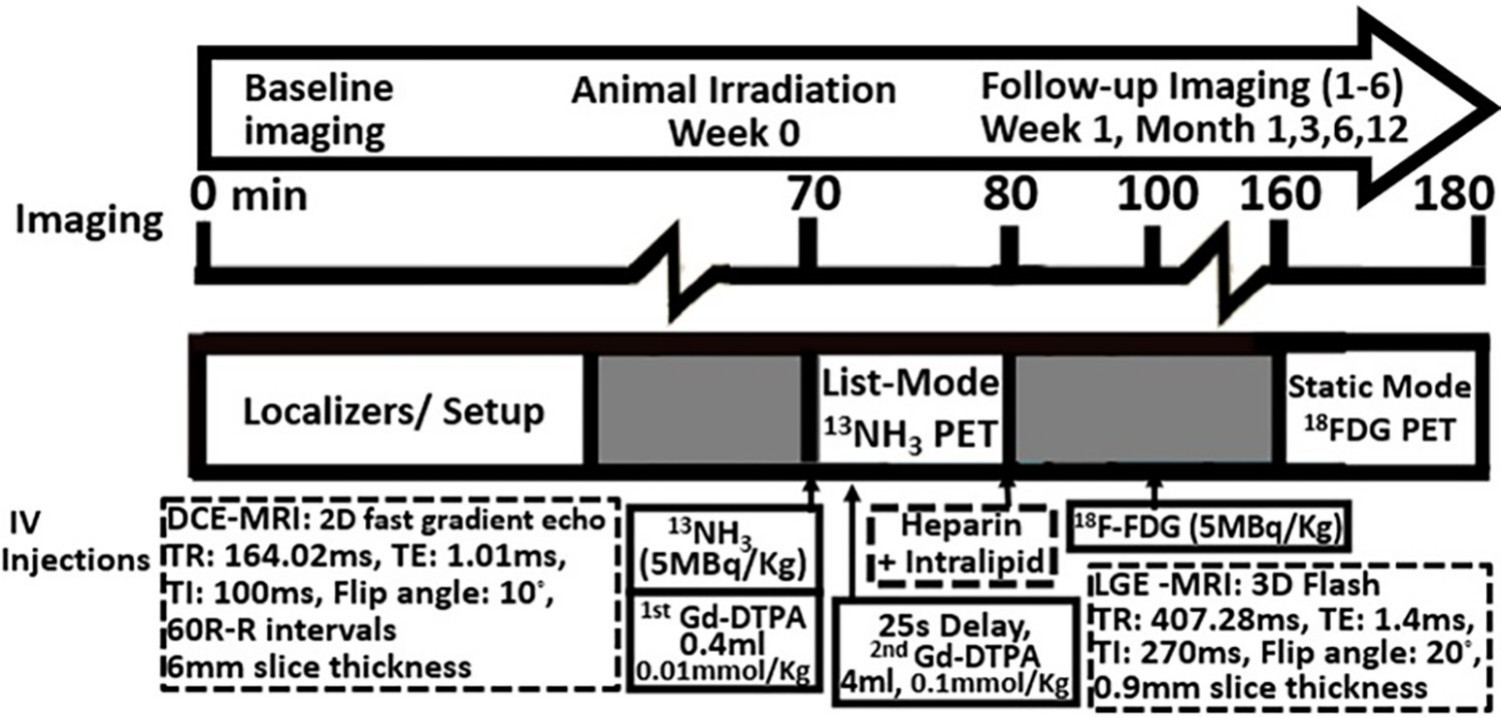
Overview of the PET/MRI imaging protocol and timing of the baseline, 1 week, 1,3,6 and 12 months follow-up imaging protocol. This protocol incudes the ^18^FDG details that have been previously reported [14].

Cardiac perfusion was longitudinally assessed using ^13^NH_3_ for the PET imaging component, along with simultaneous dual bolus dynamic contrast enhanced MR (DCE-MR) imaging using a hybrid PET/MR system (mMR, Siemens Medical Systems). Cardiac perfusion was imaged at rest. Perfusion was assessed with an injection of ^13^NH_3_ (~5 MBq/kg) (See Fig 1 for imaging protocol). The PET data was acquired in list-mode and retrospectively binned into 16 time periods 12×10s, 2×30s, 1×60s, 1×360s. All PET data was reconstructed using an iterative 3D ordered subset expectation maximization algorithm (OSEM) with 3 iterations, 21 subsets, 172×172×127 matrix size and a 4mm Gaussian smoothing filter, which yielded a voxel size of 2.08×2.08×2.03 mm.

Concurrent with ^13^NH_3_ imaging, DCE-MR imaging was performed utilizing the 2D fast gradient echo sequence (syngo MR B20P) with 6mm slice thickness, 164.02 ms repetition time, 1.01ms echo time, 100 ms inversion time, FOV matrix = 270x360, flip angle = 10° and a total scan time of 60 R-R intervals. The volume ratio of the injected Gadolinium chelate (Gd-DPTA) (Magnevist; Berlex Canada; Lachine, Quebec, Canada) was 1:10, i.e. the injection that produced the low blood concentration was 0.4 ml (0.01 mmol/kg of Gd-DTPA) and the one that produced the high blood concentration was 4 ml (0.1 mmol/kg Gd-DTPA). Each bolus was followed by 11 ml saline at a rate of 3 ml/s. The dual bolus injection was set up following the dual-bolus injection scheme stated by Ishida et al. using a two-syringe power injector with programmable pause functionality [17]. The bolus with 4 ml contrast was injected 25 seconds after the 0.4 ml bolus. T1-weighted late gadolinium enhanced whole heart imaging was performed using 3D-FLASH sequence with 407.28 ms repetition time, 1.4 ms echo time, 270 ms inversion time, FOV matrix = 250x320, slice thickness of 0.9 mm and flip angle = 20° and were collected only at 6-months and 12-months follow-up, to identify any specific focal enhancement.

The imaging protocol for ^18^FDG was adopted from a previously validated study reported by Prato et al., which is capable of identifying abnormal accumulation of inflammatory cells within the heart following coronary occlusion [15]. Glucose uptake of cardiomyocytes was suppressed in all animals through fasting and an intravenous injection of heparin immediately followed by a 20% lipid infusion, 20 mins prior to the injection of ^18^FDG. The ^18^FDG data were acquired in list mode triggered by respiration, 60 mins after the injection, using a single static frame (20 mins duration). The ^18^FDG measurements done in these animals have been published previously in which the standard uptake values remained persistently elevated compared with baseline (1.1 ± 0.03 vs. 2.6 ± 0.19, P < 0.05) [14]. The presence of myocardial inflammation was confirmed histologically through ex-vivo analysis using an anti-CD45 antibody, when the animals were sacrificed following the 1-year imaging session, euthanized using an intravenous injection of potassium chloride under anesthesia using propofol (4–6 mg/kg) [14]. However, no assessment for myocardial fibrosis was done on the pathological specimens.

### Data analysis

**Fig 2:**
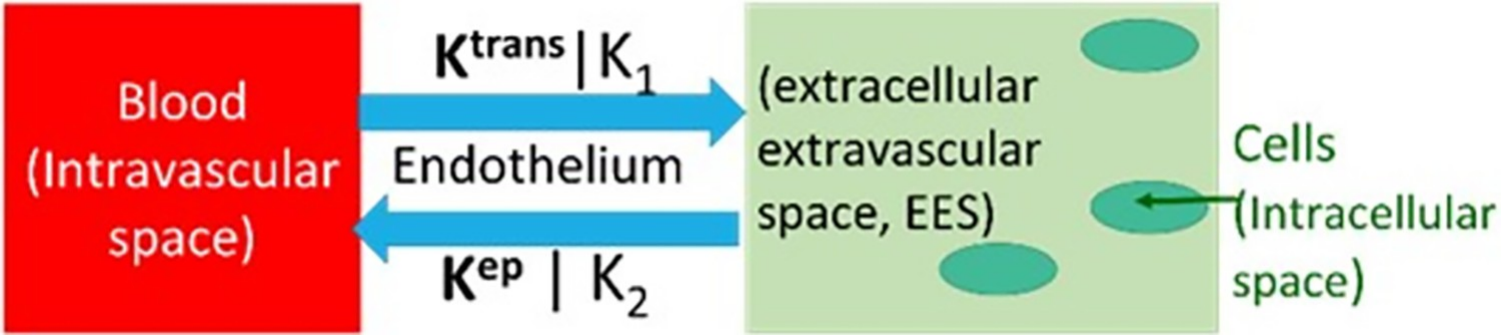
Regional uptake and clearance parameters K_1_ and k_2_ indicated in a one-tissue compartment model implemented in FlowQuant software for ^13^NH_3_ and Toft’s model with transfer constant K^trans^ and K_ep_ used by DB DCE-MRI deconvolution analysis.

Myocardial blood flow (MBF) determined from the ^13^NH_3_ injection was assessed using a one tissue-compartment tracer kinetic model of the first 4 minutes of data, as implemented in a semi-automated analysis program, FlowQuant© (University of Ottawa Heart Institute):

$C_{t}(t)=K_{1}e^{-k_{2}t}*C_{LV}(t)$, in which K_1_ [ml/min/g] and k_2_ [min^-1^], are regional uptake and clearance parameters; C_t_(*t*) and C_LV_(t) are the concentration of ^13^NH_3_ in the myocardial tissue and in the left ventricle blood. K_1_ is related to MBF according to the Renkin-Crone function: $K_{1}=\left(1-a\times e^{-\frac{b}{MBF}}\right)\times MBF$, using a and b parameters measured previously in dogs [18]. The parameters K_1_ and k_2_, are estimated parameters using a weighted least-squares algorithm [19] (See Fig 2). For ^13^NH_3_ the quantity is approximated $\left(1-a\times e^{-\frac{b}{MBF}}\right)$ ≈ 1 for MBF less than 6 ml/min/g [18].

**Fig 3:**
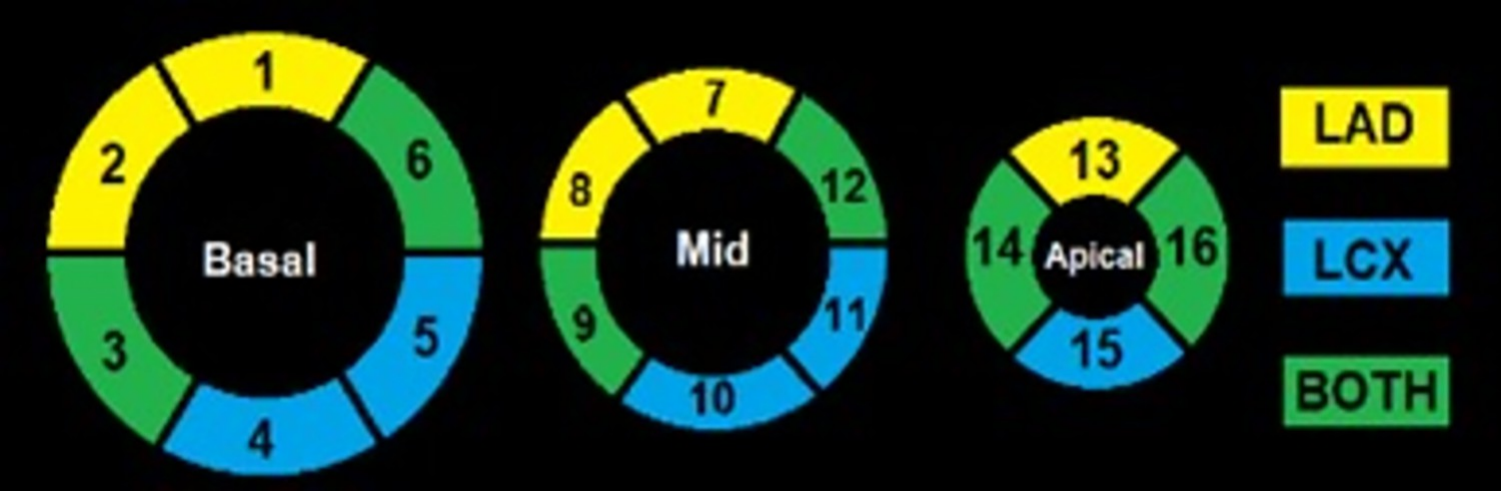
Myocardium contoured using 16-segment canine cardiac model on ITKsnap.

Three short axis slices including basal, mid and apex, of the myocardium DCE-MR images were contoured on ITK-SNAP (Version 3.6.0) [20] according to a 16-segment canine heart model (See Fig 3). The mid slice LV blood pool contour was selected as the region for the arterial input function. A MATLAB v.R2019b (MathWorks®, Natick, Massachusetts, USA) based quantitative software program was created to automate the Toft’s model (shown in Fig 2) deconvolution of the MRI derived curve fitting methods in order to calculate K^trans^. Regarding the dual bolus curve fitting method (DB), the signal intensity curve of the small bolus arterial input function (AIF) was magnified by a factor of ten according to the bolus ratio of contrast material injected. The time intensity curve and the myocardial tissue curves from the 0.4 ml bolus were truncated. The conventional 4 ml Gd-DTPA bolus myocardial tissue curves were fitted with the magnified arterial input curve using the Toft’s model: [21, 22]

$C_{t}(t-t_{0})=K^{trans}\int_{0}^{t}C_{p}(\tau )e^{-k_{ep}(t-t_{0}-\tau )}d\tau =K^{trans}e^{-k_{ep}(t-t_{0})}\int_{0}^{t}C_{p}(\tau )e^{k_{ep}\tau }d\tau$, where k_ep_ is the flux rate constant, C_t_ is the contrast concentration in the myocardial tissue and C_p_ is the blood plasma concentration derived from the AIF corrected by an assumed blood hematocrit value of 0.45. The MBF was subsequently calculated from K^trans^ obtained from each curve fitting method using the relation that equals the extraction fraction multiplied by the MBF. An extraction fraction of 0.5 was used as reported by Tong et al. for normal canine myocardium for Gd-DTPA [23].

**Fig 4:**
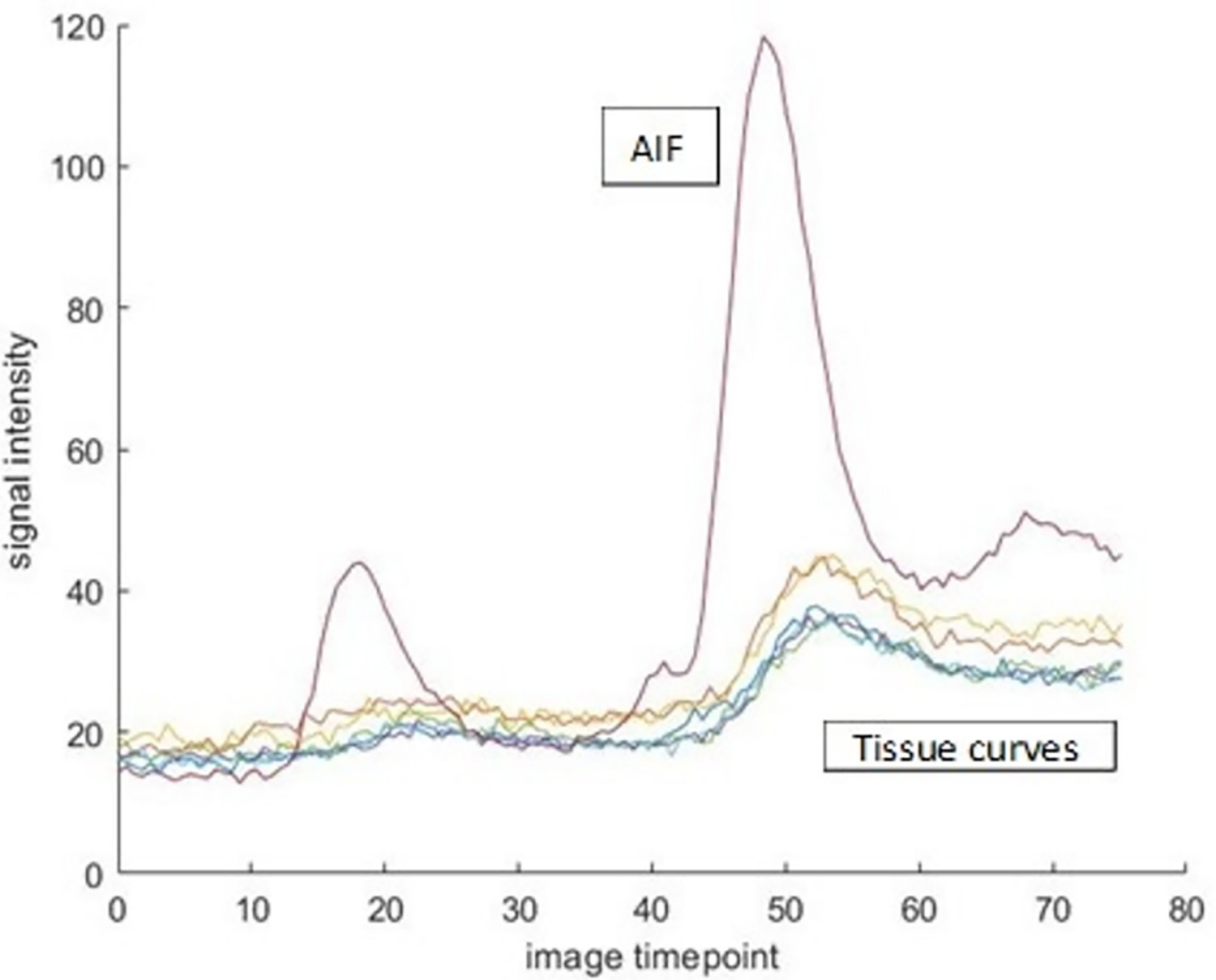
Ideal signal intensity curves for AIF and myocardial tissue within a dual bolus DCE-MR injection. Note that the low dose bolus is injected first before the high dose bolus injection.

**Fig 5:**
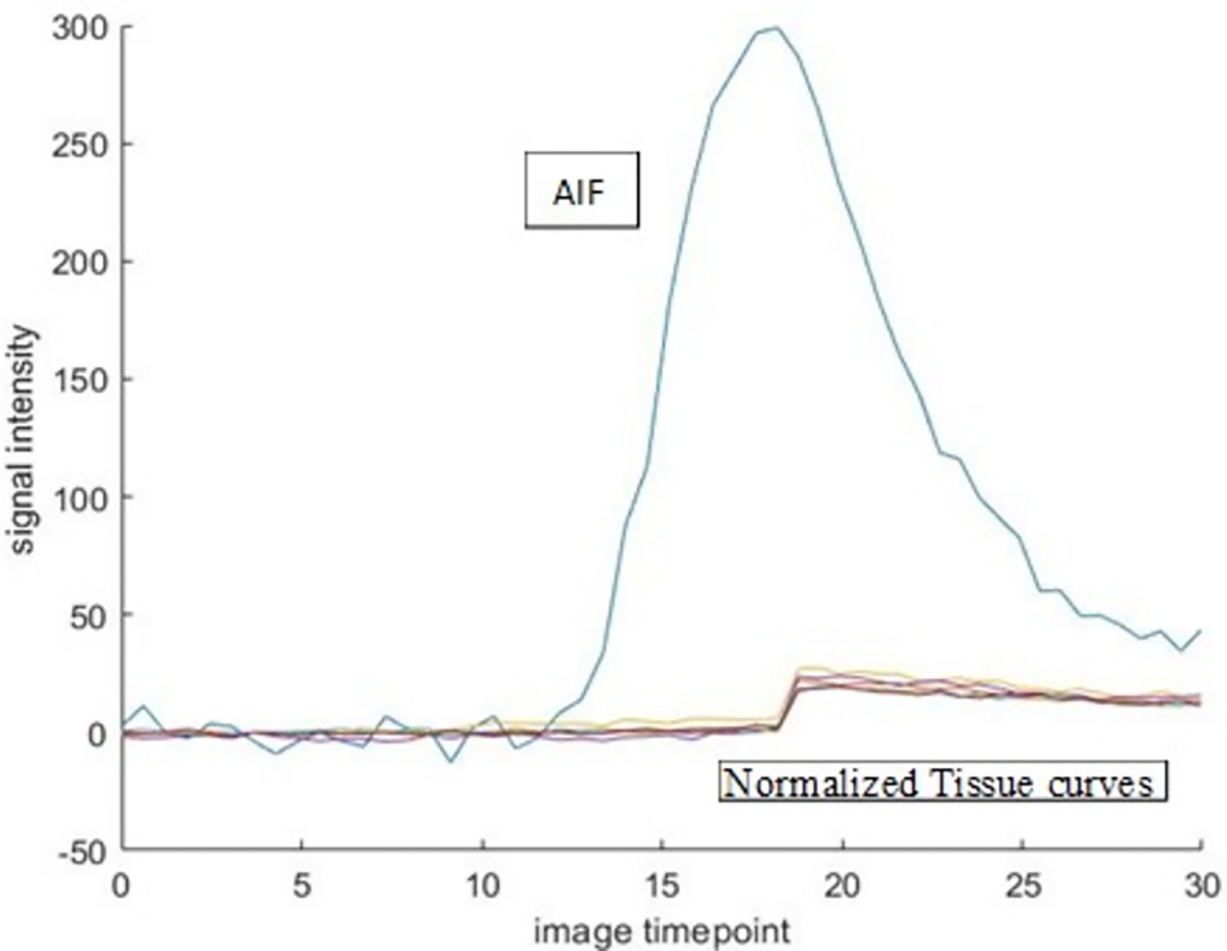
Ideal dual bolus curve fitting scenario with (a) amplification of low contrast concentration bolus AIF signal intensity curve and (b) truncation of low concentration tissue curves, followed by (c) normalization of both curves.

Figs 4 and 5 shows the ideal curve fitting scenarios for AIF and myocardial tissue curves following DB DCE-MR. Note that for both ^13^NH_3_ and DB DCE-MRI determination, a one-tissue compartment model is assumed as shown in Fig 2. Late gadolinium enhancement-MR (LGE-MR) images were analysed using circle CVI42 version 5.11 (Circle Cardiovascular Inc., Calgary, Canada). The detection of scar or fibrosis in the form of a focal enhancement, was based on the signal threshold versus reference myocardium technique (mean ± 5 SD signal intensity), with the mean obtained from a mid-slice of the left ventricle judged to correspond to non-irradiated remote myocardium [24].

### Statistical analysis

Statistical analyses were performed in SPSS IBM v.23 (IBM SPSS Statistics for Windows, Armonk, NY). The paired sample t-test determined the significance between the MBF determined from ^13^NH_3_ and the DB curve fitting technique based on coronary regions. Pearson’s bivariate correlation coefficient was used to test the association between MBF of ^13^NH_3_ and DCE-MRI using DB curves, as well as the association of ^18^FDG [14] and MBF of the two methods. Changes of MBF between ^13^NH_3_ and DB curves of DCE-MRI compared to baseline per coronary regions and changes of mean RPP were tested using non-parametric Kruskal-Wallis and Mann-Whitney test.

## Results

**Fig 6:**
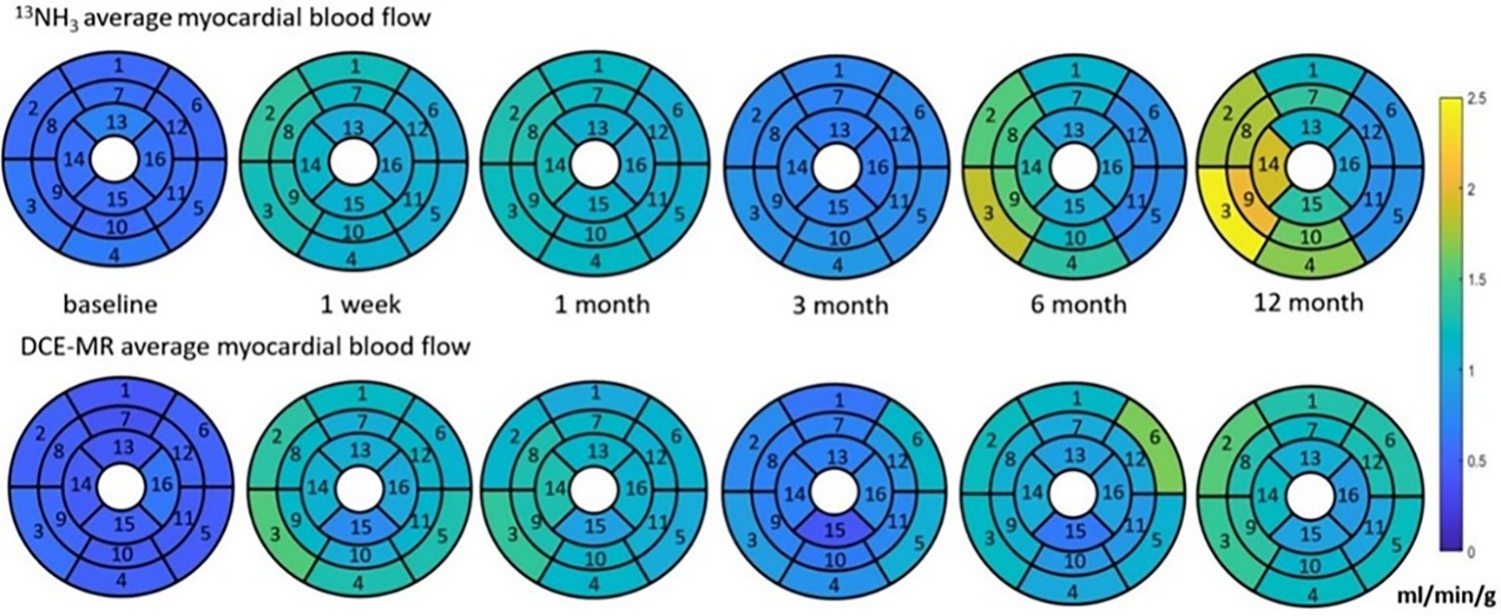
The absolute myocardial blood flow for each of the 16 segments averaged over all 5 animals. The ^13^NH_3_ results are shown at the top and the DB DCE-MRI results at the bottom.

**Fig 7:**
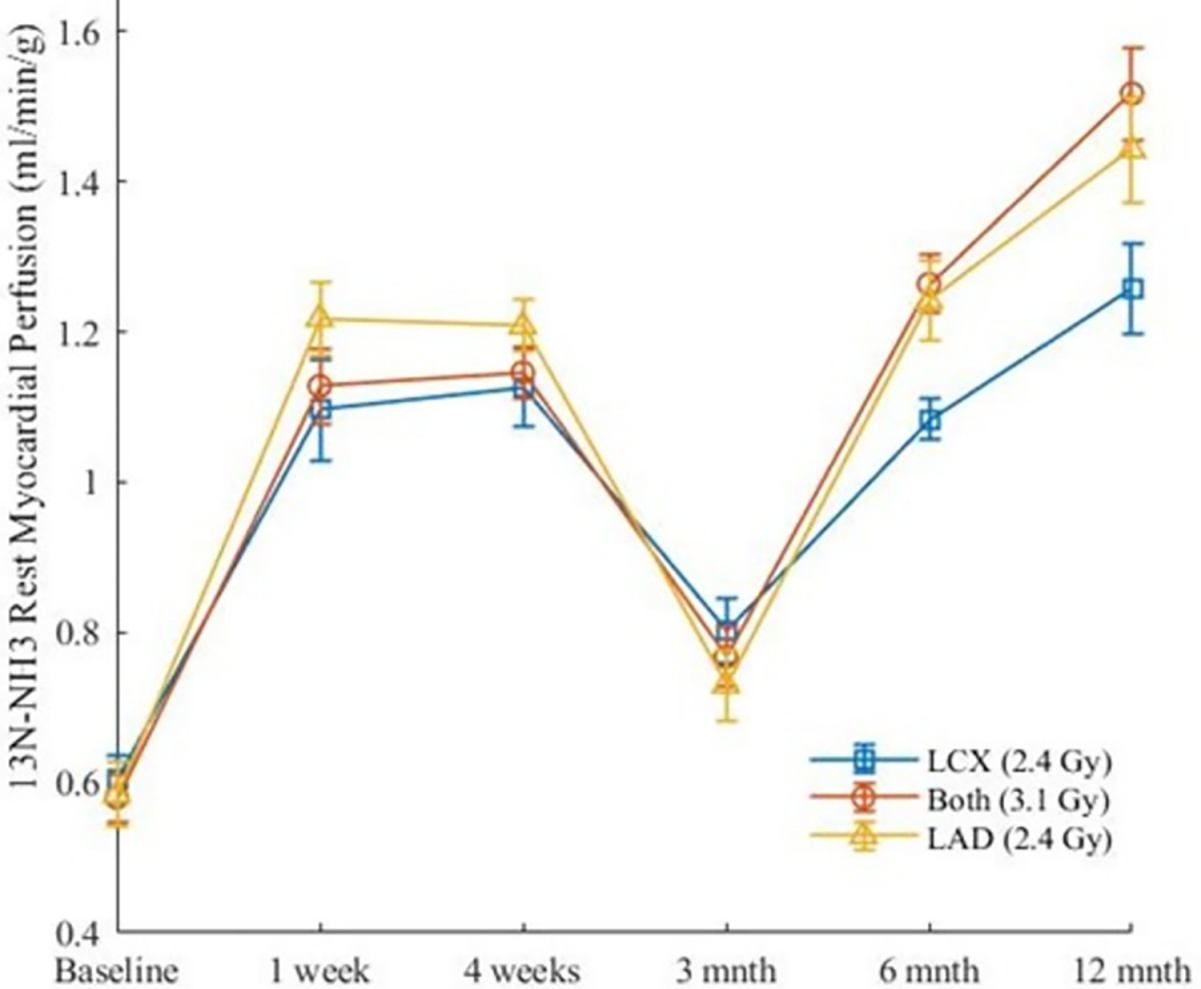
Changes and standard errors of means (SEM) in ^13^NH_3_ rest MBF based on coronary regions.

**Fig 8:**
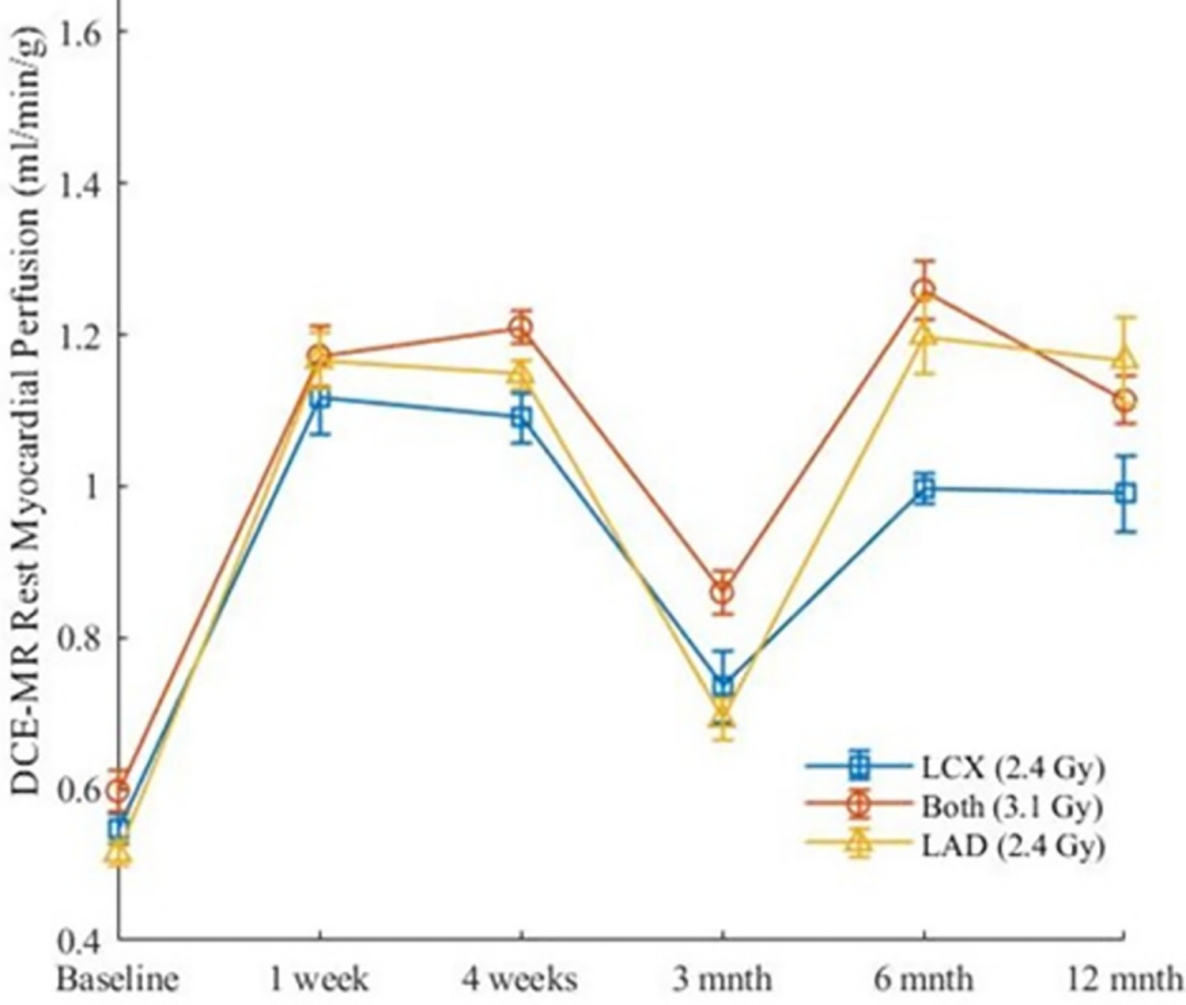
Changes and SEM in DB DCE-MRI rest MBF from DB curve fitting method based on coronary regions.

Fig 6 shows the results for both MBF methods rendered onto the 16-segments canine cardiac model (Fig 3). Figs 7, 8 show the results over time for MBF measured with ^13^NH_3_ and DB DCE-MRI respectively for the entire myocardium which can be broken down to regions supplied by the LAD, LCX and that supplied potentially by both arteries as shown in Fig 3.

**Fig 9:**
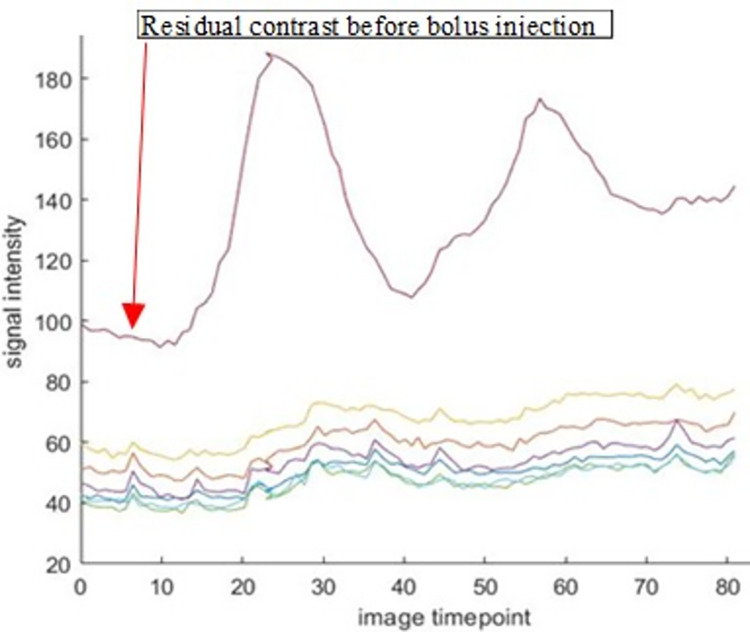
Residual contrast shown in LV pre-contrast injection of the high dose bolus injection.

**Fig 10:**
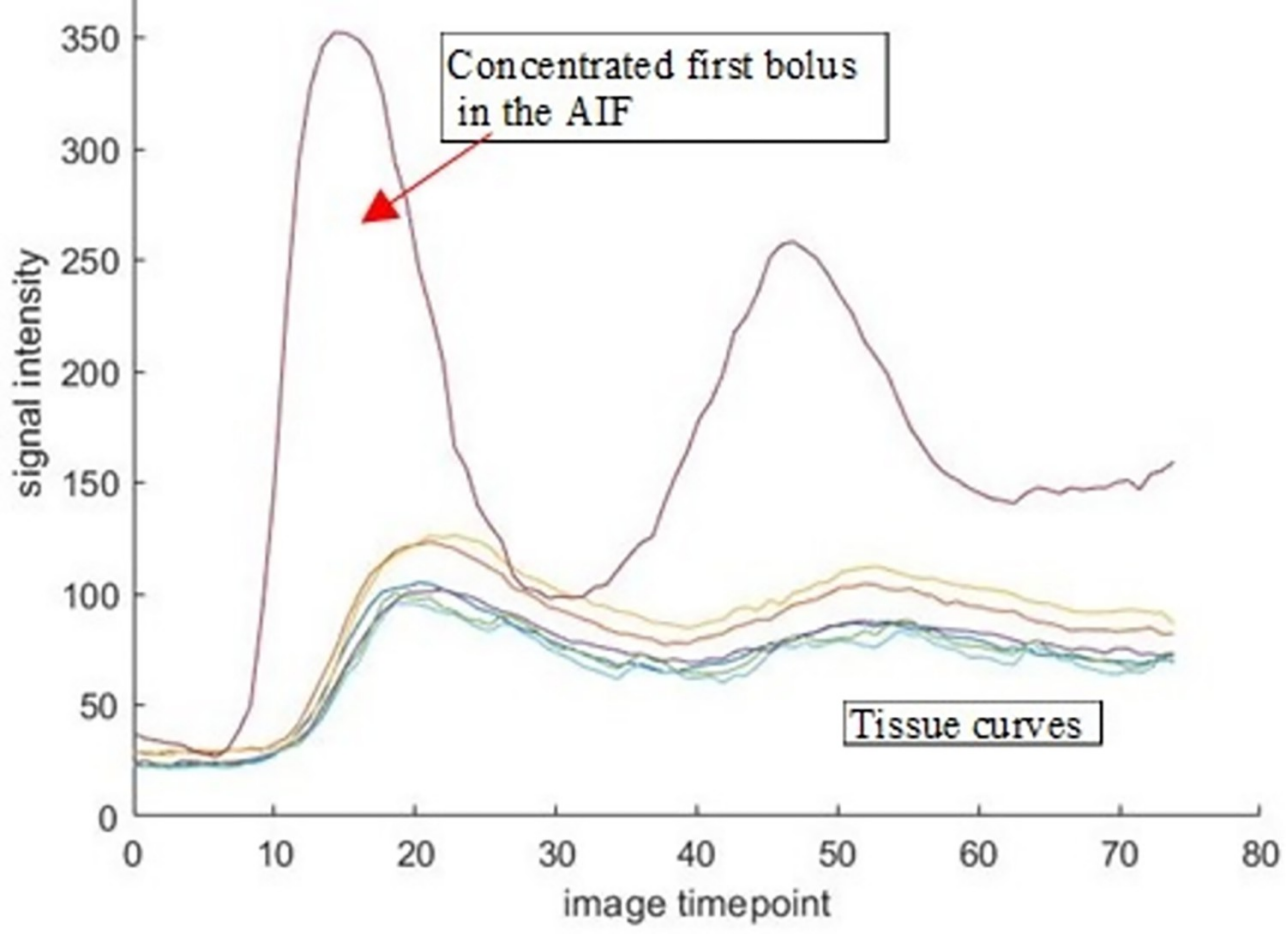
Higher blood contrast concentration following the first bolus with larger signal intensity compared to the second bolus indicating that a high dose bolus injection was injected first in error.

**Fig 11:**
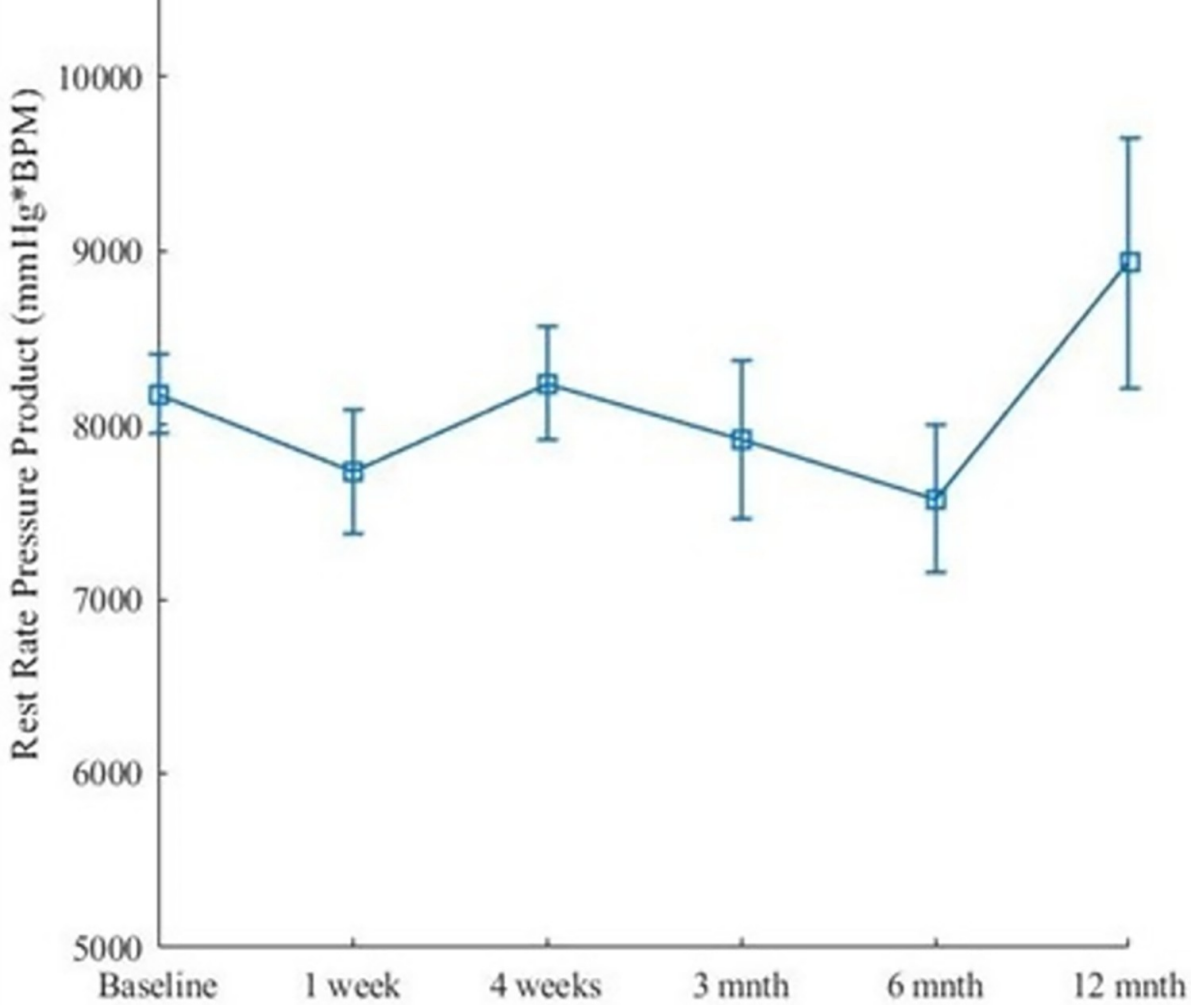
Rest rate pressure product and SEM of each imaging timepoint. Insignificant changes of mean RPP over all timepoints (p = 0.97) was determined using the Kruskal-Wallis test. Note that at 3-months in, one of the dogs, blood pressure measurements were not available.

No difference was seen when ^13^NH_3_ MBF in different coronary regions were compared to each other (p = 0.06). Significant changes developed in ^13^NH_3_ MBF in all myocardial regions for all timepoints (p <0.05) using Kruskal-Wallis test (See Table 1 column 2). Comparing MBF follow-up timepoints to baseline MBF, only the 3-months follow-up timepoint showed insignificant changes (p >0.05) in all myocardial regions (See Table 1 column 5). A positive correlation between ^18^FDG and ^13^NH_3_ MBF was significant for the LAD, LCX and that part of myocardium served by both coronary arteries (See Table 2). Note DB DCE-MRI MBF were statistically similar to ^13^NH_3_ MBF for all coronary regions (See Tables 3, 4). Note that six out of thirty data points from the DB DCE-MRI were removed from data analysis due to technical difficulties as shown in Figs 9, 10. Note that both MBF methods give, within statistical error, the same overall results: MBF post radiotherapy was increased at all timepoints measured except for the 3-months timepoint. This correlation of data from two different modalities for perfusion assessment was expected given that both were analyzed using a one-compartment tissue model (Fig 2). The ^13^NH_3_ results are highlighted given they corresponded to complete left ventricle tissue coverage, were not dependent on an assumption of extraction fraction estimate, did not fail in any of the measurements and provided overall greater statistical certainty in relationship to the ^18^FDG values. No significant changes of mean RPP over all timepoints (p = 0.97) were observed using the Kruskal-Wallis test (Fig 11). No specific focal enhancement was identified in all LGE-MR images at 6-months and 12-months follow-up.

**Table 1:** P-values of changes in time of myocardial MBF obtained from ^13^NH_3_ MBF from non parametric Kruskal-Wallis test and P-values of MBF comparing follow-up versus baseline MBF from Mann-Whitney test based on coronary regions. P < 0.05 determines significant differences.

MBF	P-value of changes in all timepoints	P-values of comparing follow-up versus baseline MBF				
		1wk	1mon	3mon	6mon	1year
^13^NH_3_ LAD	.004	.009	.009	.142	.016	.016
^13^NH_3_ LCX	.014	.016	.016	.142	.009	.032
^13^NH_3_ BOTH	.004	.016	.009	.142	.016	.016

**Table 2:** Pearson bivariate correlation coefficient and P-values of ^18^FDG standard uptake value and ^13^NH_3_ MBF based on coronary regions for all timepoints.

	r-value	P-value
^13^NH_3_ LAD MBF V.S. ^18^FDG LAD	.494	.008
^13^NH_3_ LCX MBF V.S. ^18^FDG LCX	.465	.013
^13^NH_3_ BOTH MBF V.S. ^18^FDG BOTH	.511	.005

**Table 3:** P-values and Pearson bivariate correlation coefficient of MBF obtained from DB DCE-MRI compared to ^13^NH_3_ and to ^18^FDG standard uptake values for all time points and coronary regions. R > 0.75 and R > 0.5 determines strong and moderate positive correlation respectively. Insignificant difference was shown when DB DCE-MRI MBF in different coronary regions were compared (p = 0.06).

	P-value	r-value
^13^NH_3_ LAD MBF V.S. DB LAD MBF	.057	.933
^13^NH_3_ LCX MBF V.S. DB LCX MBF	.251	.801
^13^NH_3_ BOTH MBF V.S. DB BOTH MBF	.867	.851
DB LAD MBF V.S. ^18^FDG LAD	.006	.541
DB LCX MBF V.S. ^18^FDG LCX	.175	.286
DB BOTH MBF V.S. ^18^FDG BOTH	.033	.437
DB LAD V.S. DB LCX	.063	

**Table 4:** P-values of changes in time of myocardial MBF obtained from DB-DCE MBF from non-parametric Kruskal-Wallis test and P-values of MBF comparing follow-up versus baseline MBF from Mann-Whitney test based on coronary regions. P < 0.05 determines significant differences.

MBF	P-value of changes in all timepoints	P-values of comparing follow-up versus baseline MBF				
		1wk	1mon	3mon	6mon	1year
DB LAD	.007	.021	.021	.083	.014	.034
DB LCX	.043	.021	.021	.248	.014	.034
DB BOTH	.019	.021	.021	.083	.014	.034

## Discussion

In this study, an increase of myocardial blood flow developed as early as one-week post irradiation, with a dip at 3-months follow-up during rest perfusion. The 3-months follow-up was not different compared to baseline. The biphasic response of blood flow increase was unexpected although a similar response was observed in the FDG study, i.e. there was a pause at 3 months in the increase of FDG. Although it is purely speculative, we suggest that the endothelial population in the myocardium could experience an inflammatory response before it occurs in the myocytes with small increase in resting blood flow occurring in two waves with the first due to endothelial inflammation and the second due to cardiomyocyte inflammation and fibroblast activation. This is supported by the fact that in the myocardium there are equal numbers of myocytes and endothelial cells [25]. And that endothelial cells are more sensitive to radiation injury than myocytes [26]. All other timepoints showed a significant and progressive increase over the baseline value. Note that the consistency of the MBF measures between ^13^NH_3_ vs. dual bolus DCE-MRI, i.e. two independent measures, provides assurance that the increase in MBF post radiotherapy is valid in this small group of animals. As the RPP did not change at any time point compared to baseline, the increases seen in MBF cannot be explained by changes in the conventional myocardial oxygen demand factor.

It is of interest that at 12-months ^13^NH_3_ MBF results progressed to their highest value whereas the DB DCE-MRI estimates of MBF at 12-months are similar to the other follow-up timepoints except 3-months. Due to technical issues (see Figs 9, 10), only data from 3 dogs DB DCE-MRI at 12-months were analyzed. At this 12-month timepoint, using Mann Whitney statistical analysis, only the LAD region’s MBF was significantly different between the ^13^NH_3_ and DB DCE-MRI (p = 0.03). Furthermore, comparing all follow-up timepoints, ^13^NH_3_ and DB-DCE MRI MBF were each insignificantly different except at 3-month when regional flows were compared (p ≥0.22). The marginally significant disparity of ^13^NH_3_ vs DB DCE-MRI MBF at only 12 months may be due to the intrasubject biological variations between the two modality measurements, in which DCE-MRI depends on functional capillary density, while ^13^NH_3_ depends on the glutamine synthetase reaction in the myocardial tissue. Another possibility is that the extraction fraction (EF) of Gd-DTPA may have been reduced perhaps due to loss of capillary density. As the EF of ^13^NH_3_ is not reduced until much higher flows are reached, this could be an alternative explanation. It will be of interest to determine in future work if there are corresponding changes in histology (e.g. apoptosis, fibrosis) or in T1-values (caused by increases in the extracellular volumes, but which would require T1 values to be assessed before and after contrast enhanced MRI).

In contrast to our results reported here in the canine model, clinical assessments of MBF after left-sided breast radiotherapy have consistently shown reductions in rest MBF determined primarily using SPECT imaging. However those assessments have all been at 6 or more months after radiotherapy [7–11]. The disparity between our findings and those previously reported may due to (1) the difference in human and canine’s vasculature in which the dominant coronary artery in canines is the left circumflex and not the left anterior descending; (2) the greater radiation dose deposited in the heart (up to single fraction equivalent mean dose of 2.82 Gy in literature versus 1.7 Gy in our study) and (3) the greater radiation dose deposited in the left ventricle (up to single fraction equivalent mean dose of 10.16 Gy in literature versus 2.7 Gy in our study). This greater radiation dose in the clinical SPECT studies resulted from selection criteria which followed-up primarily patients who received a high radiation dose in a portion of their left ventricle or heart during radiotherapy. In ^13^NH_3_ PET studies in the literature, Rasmussen et al. [12] reported no differences in MBF between irradiated and non-irradiated myocardium when imaging breast cancer patients at an average of 7 years post-irradiation. Note however that this study did not have baseline measures for comparison. Without our baseline measurements, our post irradiation MBF values may not be considered increased. In contrast, although Yan et al. [13] did not perform baseline measurements, they used a control group of 18 dogs for the 18 exposed animals and did ^13^NH_3_ measurements at 3, 6 and 12 months after a 20 Gy irradiation of the left anterior myocardium. No effects were seen at 3 months but a reduction in MBF was seen at 6 months with a further reduction in blood flow seen at 12 months [13]. In the same study, gradually decreasing focal areas of increased ^18^FDG uptake were observed comparing irradiated and nonirradiated canine groups at 3, 6 and 12-months due to cardiac remodeling, without differences in the proinflammation phenotype macrophage marker CD68 and inflammatory cytokines [13]. In our canine study, we were aiming to simulate typical breast cancer radiotherapy dosage which is approximately ten times lower compared to Yan et al. For our canine study we observed a significant increase of global ^18^FDG uptake which increased progressively from 3-months to 6-months and then again at 12-months. Currently, there are no serial follow-up studies post-irradiation using ^13^NH_3_ or DB DCE-MRI along with concurrent cardiac inflammation data reported except for the Yan et al. [13] study; however, as noted above, the results of that study may not be relevant to the current practice of RT in left sided breast cancer, because of the much higher administered dose in the previously reported study.

The correlation of myocardial blood flow to inflammation measured by ^18^FDG did show a significant linkage with ^13^NH_3_ myocardial blood. However, the Pearson correlation values were on average 0.5 indicating that only 25% of the change in ^18^FDG uptake was associated with changes in blood flow based upon semi-quantitative measurement. Consider specifically the 3-month time point when resting blood flow returned to baseline value, while in the ^18^FDG data, the increase at 1-month remained at 3-months. This suggests that at least part of the inflammatory signal is not mediated by changes in blood flow and is due to other additional and as yet undefined mechanisms. Also, it is not clear which is the primary driver of the pathophysiology-i.e. is the augmentation of flow an important driver of the inflammatory response, or alternatively, is the increase in flow a response to inflammatory injury. In our study, cardiac apoptotic tissue was not identified in our LGE-MR and ^18^FDG results contrary to Yan et al. [13]. The increase in rest ^13^NH_3_ MBF at all time points, save at 3-months, suggests that this could be the result of an acute global inflammation in the myocardium despite the focal radiation targeted towards primarily the LAD region [14]. The global nature of the inflammation is also consistent with the finding that no specific focal enhancement was identified from the LGE-MR images at 6-months and 12-months follow-up, consistent with the evidence of increasing global ^18^FDG uptake and increased global MBF values with a constant RPP. This global inflammatory and MBF response, seen as early as 1-week post irradiation, may initiate late fibrosis within myocardium and/or epicardial vessels. Note that any global change of scar/fibrosis would not be detected using delayed contrast enhancement, as this would require pre and post contrast T1 maps with calculation of the extracellular volumes in future studies.

**Fig 12:**
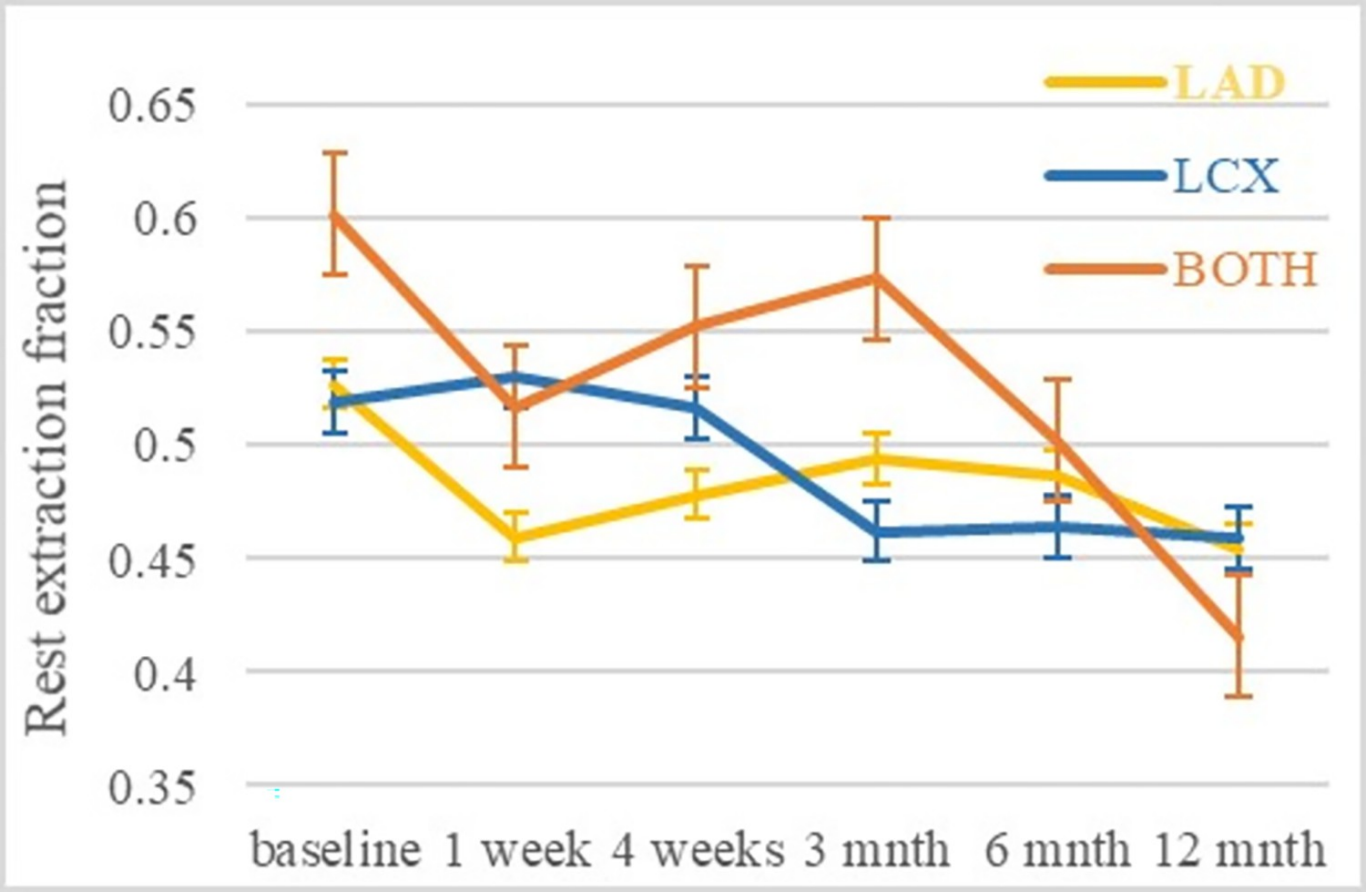
Rest extraction fraction average and SEM per coronary region determined from DB curve fitting method of DCE-MRI with K^trans^ divided by MBF from ^13^NH_3_. The extraction fraction average per coronary region for all timepoints was 0.48 ± 0.03 (mean ± standard deviation) for LAD, 0.49 ±0.03 for LCX and 0.53 ± 0.07 for both coronary regions.

A limitation of our study is the small number of canines and the lack of an independent gold standard of perfusion such as the use of microspheres [27]. However, when calculating the absolute extraction fraction with K^trans^ obtained from the DB method divided by MBF determined using ^13^NH_3_, the absolute extraction fraction average values per coronary regions obtained in this study across all timepoints (See Fig 12) were between 0.48–0.57, consistent with the value reported by Tong et al. [23] using a gold standard methodology. MBF determined from DB DCE-MRI was not statistically different from that determined from ^13^NH_3_ for all coronary regions, with a strong correlation between the two, with r values between 0.80 and 0.93 (See Table 3). An important indicator sensitive to coronary artery health [28] is myocardial perfusion reserve (MPR) which corresponds to the ratio of myocardial blood flow at stress divided by that at rest [29]. Although not assessed in our study, this would be a valuable measure to have in future canine and human studies. However, stress blood flow measurement requires pharmacological intervention. Hence, if only rest MBF along with FDG are needed, clinical adoption will be better facilitated. Moreover, the PET (voxel size of 2.08×2.08×2.03mm) and contrast enhanced MRI (voxel size of 1.875×1.875×1mm) in our canine study did not have the needed resolution in order to identify the transmural distribution of changes in flow (i.e. subendocardial vs. subepicardial). A possible confound is that the dogs were a year old when their hearts were irradiated while for women the age is typically 40 plus years. Given the life expectancy for hounds is about 10 years the irradiation was given when 10% into their life cycle, while for humans it is during the last half of their life cycle. Although findings in this young canine model have an excellent track record in translation to older human patients in the area of myocardial infarction progression [30, 31] translating effects of cardiac radiation injury has not been demonstrated. One consideration is that in humans, cardiomyocytes renew approximately 1% per year at age 20 and this decreases with age to 0.3% by age 75 [32]. If the effect of radiotherapy is to preferentially destroy the dividing myocyte stem cells, then there could be a more accelerated effect when younger individuals are irradiated. This could explain the long latency period between irradiation and subsequent heart damage reported in humans. Although we are not aware of similar data in dogs regarding myocyte regeneration, it is reasonable to expect a shorter myocyte regeneration time in young dogs compared to the relatively older human breast cancer patients. Nevertheless, the underlying pathology i.e. initial inflammation, followed by fibroblast activation and fibrosis, has been shown to be similar in dogs and humans [33]. Hence, it is reasonable to suggest that the canine model is a good model for human cardiac damage due to radiotherapy. Although the timing of blood flow and inflammation change may be different.

## Conclusion

In the canine, rest myocardial blood flow within the first year following heart irradiation generally progressively increases over time. This has been confirmed by two non-invasive independent methods. A possible interpretation is that the increase in resting MBF is a response to myocardial inflammation. Based on this data, future patient studies early after radiotherapy, should consider measurements of both myocardial blood flow and myocardial inflammation.

## Supporting information

S1 Data (XLSX)
